# Spatial analysis of photoplethysmography in cutaneous squamous cell carcinoma

**DOI:** 10.1038/s41598-022-10924-3

**Published:** 2022-05-05

**Authors:** Simon Mylius Rasmussen, Thomas Nielsen, Henrik Hager, Lars Peter Schousboe

**Affiliations:** 1 Department of Otolaryngology, Southdanish University Hospital, 7100 Vejle, Denmark; 2 Department of Electrical and Computer Engineering, Aarhus University, 8000 Aarhus N, Denmark; 3 Department of Clinical Pathology, Vejle Hospital, 7100 Vejle, Denmark; 4 Department of Regional Health Research, University of Southern Denmark, 5000 Odense, Denmark

**Keywords:** Surgical oncology, Biomedical engineering

## Abstract

The primary treatment of the common malignancy squamous cell carcinoma is surgical removal. In this process, sufficient tissue removal is balanced against unnecessary mutilation. We recently presented a remote photoplethysmography algorithm, which revealed significant differences between processed video recordings of cancer biopsy areas and surrounding tissue. The aim of this study was to investigate whether spatial analyses of photoplethysmography data correlate with post-excision pathological analyses and thus have potential to assist in tumour delineation. Based on high speed video recordings of 11 patients with squamous cell carcinoma, we examined different parameters derived from temporal remote photoplethysmography variations. Signal characteristics values in sites matching histological sections were compared with pathological measures. Values were ranked and statistically tested with a Kendall correlation analysis. A moderate, negative correlation was found between signal oscillations and the width and transversal area of squamous cell carcinoma in the frequencies below 1 Hz and specifically from 0.02 to 0.15 Hz. We have presented a correlation between frequency content and prevalence of cancer based on regular video recordings of squamous cell carcinoma. We believe this is supported by published findings on malignant melanoma. Our findings indicate that photoplethysmography can be used to distinguish SCC from healthy skin.

## Introduction

### Squamous cell carcinoma and surgical challenges

Squamous cell carcinoma (SCC) is the predominant^1^ type of head and neck cancer, and the second most common form of cutaneous malignancy^2^. Surgical excision is the main treatment modality for SCC. Histology is performed to confirm the diagnosis, and complete removal is secured by particular examination of the resection margins^2^. The complete resection of SCC in the head and neck relies on palpation and visual inspection^3^. The surgeon has to balance the need for complete tumour removal against the desire to avoid unnecessary mutilation^2^.

Increased blood supply through angiogenesis is a well known cancer characteristic^4,5^. Angiogenesis is also known and studied in SCC^6^, where vascularity differs from other non-SCC lesions, such as basal cell carcinoma and melanoma^7–9^. An intra-operative imaging tool could help the surgeon visualise and guide the excision of the tumour with better margin control in real time^3^.

### Remote photoplethysmography

Remote photoplethysmography (rPPG) is a technique of contact-less monitoring of human cardiac activities by detection of pulse-induced subtle colour variations on human skin surface using a multiwavelength RGB camera^10^. An rPPG system typically consists of a light source shining on the skin and an RGB camera sensor recording the reflected signal^10^. The green light channel is typical of interest as it contains the strongest PPG signal, based on the fact that haemoglobin absorbs green light better than red light and penetrates deeper into the skin than blue light^11^. Because of this, blood flow is best to study through the green colour channel data.

In a previous study of SCC, we presented a video processing algorithm extracting a perfusion measure, the perfusion index, from rPPG data. The perfusion index differed significantly between the biopsy area and healthy surrounding tissue encouraging further investigation to clarify how detailed distinctions can be made^12^.

rPPG is closely associated with cutaneous perfusion and sensitive to autonomic nervous activity^13^. A correlation of 0.91 has been shown between PPG and one of the main technologies for studying micro-circulation, laser Doppler flowmetry, for endothelial activities^14^.

### Spatial analysis of cancer margins

A successful spatial analysis of cancer margins may lead to more precise excision borders. Different methods have been tested for spatial identification of cancer: Using MR perfusion imaging, blood flow has been shown to be higher in SCC than in benign tissue^15^. Near-infrared (NIR) fluorescence imaging has been demonstrated to be a sensitive and specific method of guiding surgery for head and neck cancers and potentially other cancers with challenging imaging conditions^16^. A sophisticated margin visualisation based on machine learning and fluorescence lifetime imaging has been tested and found successful, however focused on examining the excised tissue^17^.

### Vasomotion frequency characteristics in cancer and healthy tissue

The micro-vasculature in the skin has been revealed to contain at least six distinct oscillatory components, attributable to different physiological functions: Interval I (0.6–2 Hz) related to cardiac activity, interval II (0.145–0.6 Hz) related to respiratory activity, interval III (0.052–0.145 Hz) related to microvessel smooth muscle cell activity, interval IV (0.021–0.052 Hz) related to micro-vessel innervation, and intervals V and VI (0.0095–0.021 Hz and 0.005–0.0095 Hz, respectively) related to endothelial activity, both nitric oxide (NO) dependent and independent^18^.

In a malignant tumour, the vasculature is different from healthy tissue^19^. Temporal variations in perfusion, specifically oscillatory components around 0.12 Hz is higher in melanoma than in healthy tissue, while the level of frequencies in the range 0.01–0.08 Hz is increased in healthy tissue compared with cancer tissue^18^, meaning cyclic variations in the frequency range 0.01–0.08 Hz are more prevalent in healthy tissue than in melanoma tissue.

### Aim

The aim of the study was to investigate whether rPPG variations obtained from video recordings of SCC statistically correlate with the spatial region of the tumour.

## Results

The results with significant correlations above 0.34 or below −0.34 can be seen in Table 1. The levels of 0.34 and −0.34 have been set to present the best correlations. In the frequency range 0.02–0.15 Hz, there was a correlation of −0.35 between flow and the width of cancer for section widths of 2, 3, 4 and 6 pixels, suggesting cyclic variations in the frequency range 0.02–0.15 Hz are more prevalent in lesser cancerous tissue. Phase shift versus width also showed a signficant correlation of around −0.34 for frequencies below 1 Hz. Phase shift versus the tranversal area of the cancerous tissue was found to show a −0.4 correlation for frequencies below 1 Hz. The standard deviation versus the transversal area of the tumour showed a correlation of 0.34.

**Figure 1:**
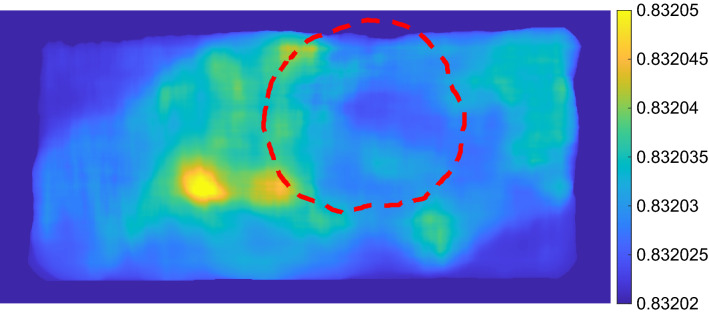
Example of chart based on the flow measure for the frequencies 0.02–0.08 Hz. Resection mask is shown with red dashed line.

**Figure 2:**
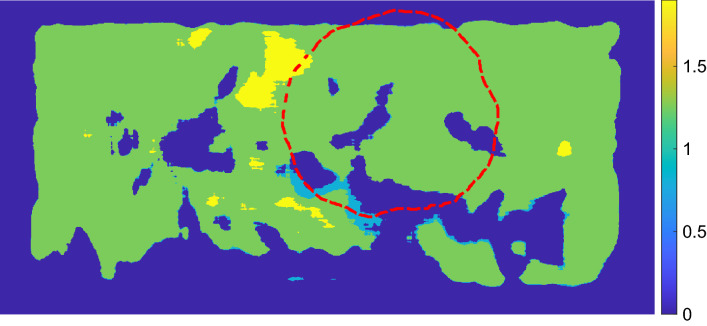
Example of chart based on the phase shift measure for the frequencies 0.02–0.08 Hz. Resection mask is shown with red dashed line.

Figures 1 and  2 show example charts of the flow measure and phase measure, respectively. From the figures, a low level of flow and phase shift can be seen inside the biopsy resection area (marked with a red dashed line).

**Table 1:** The results of Kendall correlation analysis. *Compared:* Signal characteristics vs. pathology measures. *Width:* Width of the digital section marking. *Bandpass (Hz):* Frequency spectrum analysed. *Rho:* Correlation coefficient. *P:* *P*-value.

Compared	Width	Bandpass (Hz)	Rho	P
Flow vs. width	2	0.08<f<0.15	−0.35	0.01
Flow vs. width	3	0.08<f<0.15	−0.34	0.01
Flow vs. width	3	0.02<f<0.08	−0.35	0.01
Flow vs. width	4	0.08<f<0.15	−0.35	0.01
Flow vs. width	4	0.02<f<0.08	−0.34	0.01
Flow vs. width	5	0.08<f<0.15	−0.34	0.01
Flow vs. width	5	0.02<f<0.08	−0.34	0.01
Flow vs. width	6	0.08<f<0.15	−0.35	0.01
Flow vs. width	6	0.02<f<0.08	−0.34	0.01
Flow vs. width	7	0.02<f<0.08	−0.34	0.01
Flow vs. width	8	0.02<f<0.08	−0.34	0.01
Flow vs. width	9	0.02<f<0.08	−0.34	0.01
Flow vs. width	10	0.02<f<0.08	−0.34	0.01
Phase shift vs. width	1	f<1	−0.34	0.01
Phase shift vs. width	1	f<2	−0.35	0.01
Phase shift vs. width	1	f<5	−0.36	0.01
Phase shift vs. width	2	f<1	−0.34	0.01
Phase shift vs. width	2	f<2	−0.34	0.01
Phase shift vs. width	2	f<5	−0.34	0.01
Phase shift vs. width	3	f<1	−0.34	0.01
Phase shift vs. width	3	f<2	−0.34	0.01
Phase shift vs. width	3	f<5	−0.34	0.01
Phase shift vs. width	8	f<1	−0.34	0.01
Phase shift vs. width	8	f<2	−0.34	0.01
Phase shift vs. width	8	f<5	−0.34	0.01
Phase shift vs. width	9	f<1	−0.34	0.01
Phase shift vs. width	9	f<5	−0.34	0.01
Phase shift vs. area	1	f<1	−0.39	<0.005
Phase shift vs. area	1	f<2	−0.40	<0.005
Phase shift vs. area	1	f<5	−0.40	<0.005
Phase shift vs. area	2	f<1	−0.39	<0.005
Phase shift vs. area	2	f<2	−0.39	<0.005
Phase shift vs. area	2	f<5	−0.39	<0.005
Phase shift vs. area	3	f<1	−0.40	<0.005
Phase shift vs. area	3	f<2	−0.40	<0.005
Phase shift vs. area	3	f<5	−0.40	<0.005
Phase shift vs. area	4	f<1	−0.38	<0.005
Phase shift vs. area	4	f<2	−0.38	<0.005
Phase shift vs. area	4	f<5	−0.38	<0.005
Phase shift vs. area	5	f<1	−0.40	<0.005
Phase shift vs. area	5	f<2	−0.40	<0.005
Phase shift vs. area	5	f<5	−0.39	<0.005
Phase shift vs. area	6	f<1	−0.40	<0.005
Phase shift vs. area	6	f<2	−0.40	<0.005
Phase shift vs. area	6	f<5	−0.40	<0.005
Phase shift vs. area	7	f<1	−0.40	<0.005
Phase shift vs. area	7	f<2	−0.40	<0.005
Phase shift vs. area	7	f<5	−0.40	<0.005
Phase shift vs. area	8	f<1	−0.40	<0.005
Phase shift vs. area	8	f<2	−0.39	<0.005
Phase shift vs. area	8	f<5	−0.39	<0.005
Phase shift vs. area	9	f<1	−0.40	<0.005
Phase shift vs. area	9	f<2	−0.39	<0.005
Phase shift vs. area	9	f<5	−0.39	<0.005
Phase shift vs. area	10	f<1	−0.39	<0.005
Phase shift vs. area	10	f<2	−0.39	<0.005
Phase shift vs. area	10	f<5	−0.39	<0.005
Standard deviation vs. area	2	f<1	0.34	0.01

## Discussion

The low-frequency rPPG content was significantly and inversely correlated with the width and area of cancer, and the absence of low-frequency vascular signal corresponds to the findings by Lancaster et al.^18^ using laser Doppler flow measurements of melanomas.

These low frequencies likely correspond to B waves (0.008–0.03 Hz)^20,21^ and Mayer waves (0.05–0.15 Hz)^22,23^.

B waves are cyclic variations in intracranial pressure, which are related to arterial blood pressure variations through mechanisms not fully understood. Mayer waves are cyclic variations in arterial blood pressure, which have been correlated to oscillations in sympathetic nervous activity, baroreflectory control, and endothelium-derived nitric oxide, and thus peripheral vascular resistance, but the physiology is not fully understood^22,24^. Direct correlation between Mayer waves and blood vessel diameter has been demonstrated in retinal blood vessels^25^. Kiselev et al. found that low-frequency variations in PPG exists during cardioplegia and extracorporal circulation suggesting that the coupling from vessels to heart is neurogenic, but the coupling from heart to vessels is haemodynamic through cardiac output^24^.

The poorly functioning tumour vasculature may lack nervous and endogenous regulation^26^ and as a consequence not provide the low-frequency rPPG content. This may explain the observed inverse correlation between low-frequency rPPG content and width and area of cancer; with increasing width and area of cancer, an increasing proportion of the rPPG signal is of cancer origin due to the limited penetration of the optical method, and thus the rPPG signal has a decreasing content of the physiological low-frequency content. The frequencies of Mayer waves are relatively stable within species^22^, which is beneficial in refining the analysis.

The significant correlations of standard deviation vs. area for frequencies below 1 Hz could possibly be explained by chaotic flow patterns in the tumour vasculature including transient stoppages of flow in blood vessels^26^.

An advantage of rPPG is the relatively high and well-defined spatial resolution, which potentially yields parametric maps of information relevant in tumour delineation.

If these findings can be confirmed, regular video recordings of cancer might contain information on the spread of cancer tissue. By plotting a statistical measure of a correlation, calculated throughout the pixel array, the probability of cancer infiltration might be visualised for intraoperative use and could prove a valuable measure to be used in an assisted medical technology. However, the current data and results presented do not prove that such a tool increases the accuracy during surgery, which another clinical study should investigate.

The experimental setup and camera equipment could be optimised regarding sensor quality and stability conditions in the operating theatre and could maybe reveal even better results.

The most intuitive assistance tool would be a real time device for use during surgery. The fact that the findings presented in this paper are in frequencies that oscillates once to twice per minute imply a delay in the ability for real time application, to the best of the authors knowledge.

## Conclusion

We have presented a correlation between rPPG frequency components inside a SCC and the spatial prevalence of cancer measured histologically. We believe this is supported by published findings on malignant melanoma. Our findings indicate that PPG can be used to distinguish SCC from healthy skin.

## Methods

### Data and setup

The data acquisition protocol is the same as described in our recent study^12^, where additional details are described.

Our study was conducted at the Department of Otolaryngology (Hospital Lillebaelt) in Vejle, Denmark. It was authorised by the University of Southern Denmark and approved by the Danish National Committee on Health Research Ethics and in accordance with the Helsinki Declaration. We chose to focus on SCC patients exclusively since finding a pattern in the rPPG changes might be more difficult if data represents multiple pathologies. Patients referred to hospital under suspicion of SCC and patients with biopsy confirmed SCC were enrolled in the study. Twenty-one adults gave written consent to participate. Ten patients were excluded because of histologically dis-confirmed SCC or the recordings were unusable, see Table 2 for characteristics of the selected patient group. Clinically relevant information about the volunteers was logged. We recorded the skin tumour in a recording of 60 seconds.

**Table 2:** Patient characteristics.

Nr	Age	Gender	SCC location
1	82	Male	Scalp
2	63	Female	Upper arm
3	53	Female	Lower leg
4	64	Male	Scalp
5	84	Male	Finger
6	89	Male	Back of hand
7	81	Male	Temple
8	89	Female	Finger
9	75	Male	Lip
10	85	Male	Ear
11	77	Female	Scalp

For video recording we used a mobile recording system consisting of an RGB camera (UI-3160CP-C-HQ Rev2.1 sensor, iDS, Germany) and a zoom lens (Navitar Zoom 7000, Navitar, USA). Recordings were done in 12 bit, 460 $\times$ 960 pixels, 60 frames per second (fps) and for 1 minute. For more details see our recent study^12^.

The pathologist registered the tumour width, depth and area of each histological section.

### Case selection

The excised tumours were evaluated by a pathologist as to whether the tumour was SCC. Eight patients without SCC were excluded and two were excluded as the recording were not sufficient for later determination of sectional cuts.

### Video processing and registration

The video processing is mostly the same as described in our recent study^12^, where further details are described. Differences are described below.

Data were analysed in a raw uncompressed format.

Based on the reasons given in the Section on *Remote photoplethysmography*, processing and analysis were focused on data from the green colour channel. The remaining frames were registered by affine registration for the green colour channel using the Matlab rutines, imregconfig and imregister (MATLAB. (2020). version 9.8.0.1396136 (R2020a). Natick, Massachusetts: The MathWorks, Inc.). The DC offset was removed from the green colour channel data of each pixel.

### Frequency analysis

The overall idea of the frequency analysis was to find signal characteristics that correlated with the growth of SCC. In essence, each histological specimen was divided into blocks and each block had the depth, width and transversal area of the tumour growth registered. Each section cut in the histological specimen was digitally marked in the respective video recording, allowing for signal analysis of specific pixels representative of the respective histological section cut. By several different signal analysis methods, the findings from each method were correlated to the known pathological tumour growth.

**Figure 3:**
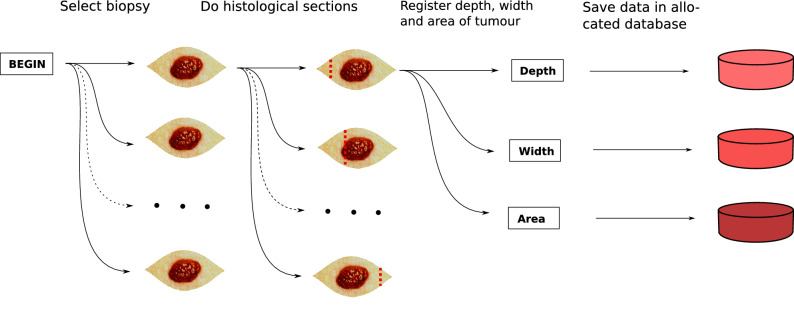
Diagram showing the acquisition of histological data in form of depth, width and area of the cancer tissue for each section cut for each biopsy.

To investigate the correlation between rPPG signal characteristics and tumour region, a correlation analysis was done between pathology variables and signal variables. Signals were measured from the area of each section cut and consisted of a combination of three variables: pixel width of section cut, frequency band and a signal characteristic, see Table 3. The pathology variables consisted of tumour width, tumour depth and tumour area. Each biopsy was cut in sections, and each section had the tumour depth, width and area registered and saved in an allocated database (see Fig. 3).

The correlation analysis was done between all combinations of signal variables and pathology variables.

**Table 3:** Signal variables in form of widths of the masks representing section cuts, the frequency bands used for band-pass filtering and the signal characteristics extracted.

**(a) Mask widths**	
Mask widths in pixels	
1	
2	
3	
4	
5	
6	
7	
8	
9	
10	

**(b) Frequency bands**	
Frequency bands in Hz	
0.08 Hz<f<0.15 Hz	
0.02 Hz<f<0.08 Hz	
0.02 Hz<f<0.15 Hz	
0.02 Hz<f<0.05 Hz	
0.05 Hz<f<0.15 Hz	
f<1 Hz	
f<2 Hz	
f<5 Hz	
1 Hz<f<10 Hz	
2 Hz<f<10 Hz	
5 Hz<f<10 Hz	
10 Hz<f<50 Hz	
20 Hz<f<50 Hz	
30 Hz<f<50 Hz	
40 Hz<f<50 Hz	

**(c) Signal characteristics**	
Signal characteristics	
Flow measure	
Phase shift	
Differentiation	
Magnitude of most prevalent frequency	
Standard deviation (SD)	
SD of the differentiation	

#### Histological examination

Each surgical specimen was fixed in 4% neutral buffed formaldehyde. The specimen was divided into blocks (see red dotted lines in Fig.  3) varying from 2 to 10 (mean of 6.6) depending on the pathologist. The blocks were dehydrated and embedded into paraffin blocks. From each block, 3 micrometre sections were cut using a microtome, Thermo scientific HM355S, Thermo Fisher Scientific, USA. After rehydration the sections were stained with haematoxylin and eosin, dehydrated, and mounted with cover slip. The sections were scanned using a Hamamatsu NanoZoomer ZR, Hamamatsu Photonics, Japan. Using NDPI view software each section was analysed for depth, width and transversal area of the tumour growth, although some sections revealed no tumour tissue. See Fig. 3.

#### Calculation of signal variables

**Figure 4:**
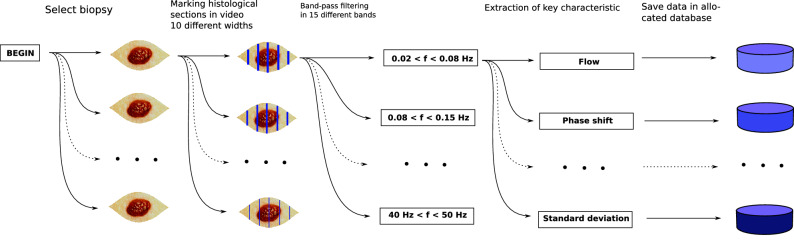
Diagram showing the acquisition of measured data, in form of extracting data from different widths of the digital section masks, band-pass filtering and extraction of the key signal characteristic.

The process of collecting data for signal variables can be seen in Fig. 4.

**Histological section area**

Although image registration had been completed, minimal movement across the pixel array may occur. To account for this, the analysis was done for histological section area widths of 1 to 10 pixels, see Table 3a and Fig. 3. So, data was extracted from 10 different pixel widths for each histological section.

**Frequency bandpass filters**

Of all extracted sectional data, all were bandpass filtered in 11 different bandpass filters to investigate the importance of frequency components, see Table 3b and Fig. 3.

**Signal characteristics**

Signal characteristics were extracted from each version of frequency filtration and saved in an allocated database, which were specific for that certain combination of histological area width, bandpass filter and signal characteristic.

We investigated the importance of six different signal characteristics:

1. **Flow measure:** We have in a recent publication documented this algorithm. It depends on the average and extremes of the signal^12^.
2. **Phase shift:** The degree of shift in oscillation with respect to a certain time point.
3. **Differentiation:** The numeric difference between a temporal sample point in a signal and the following temporal sample point.
4. **Magnitude of most prevalent frequency:** Each videopixel was analysed by fast fourier transform to reveal the frequency components of the signal. The highest amplitude was registered and averaged for the histological section area.
5. **Standard deviation:** Standard deviation of the signal.
6. **Standard deviation of the differentiation:** Simply the standard deviation of the differentiated signal.

The choice was based on a combination of our previous experiments^12^, published findings (see Section on *Vasomotion frequency characteristics in cancer and healthy tissue*) and our intuition.

### Statistics

#### Correlation analysis

**Figure 5:**
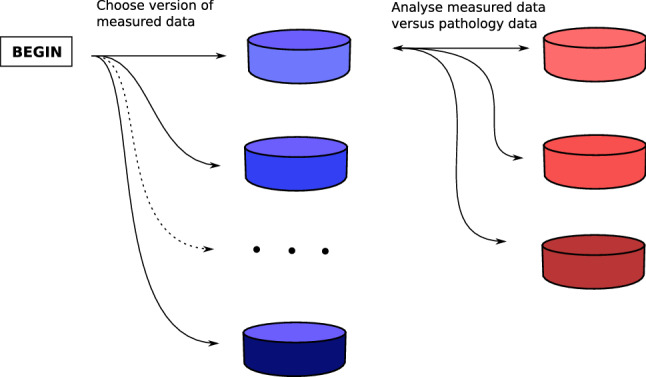
Diagram showing the correlation analysis between each combination of measured data and pathology data.

The combined signal variables (section area, frequency band and signal characteristic) was analysed vs. the pathology variables (width, depth and area of cancer) by a Kendall correlation analysis, see Fig. 5. *P*<0.05 was considered statistically significant.
